# Ghrelin Ameliorates Traumatic Brain Injury by Down-Regulating bFGF and FGF-BP

**DOI:** 10.3389/fnins.2018.00445

**Published:** 2018-07-05

**Authors:** Xuefei Shao, Qianxin Hu, Sansong Chen, Qifu Wang, Pengcheng Xu, Xiaochun Jiang

**Affiliations:** Department of Neurosurgery, Yi-Ji Shan Hospital of Wannan Medical College, Wuhu, China

**Keywords:** ghrelin, traumatic brain injury, neuroprotective effects, bFGF, FGF-BP

## Abstract

Traumatic brain injury (TBI) is a primary cause of disability and mortality. Ghrelin, a gastrointestinal hormone, has been found to have protective effects for the brain, but the molecular mechanism of these neuroprotective effects of ghrelin remains unclear. In this study, an electronic cortical contusion impactor was used to establish a rat TBI model and we investigated the effect of ghrelin on brain repair by neurological severity score and histological examination. An antibody array was employed to uncover the molecular mechanism of ghrelin’s neuroprotective effects by determining the alterations of multiple proteins in the brain cortex. As a result, ghrelin attenuated brain injury and promoted brain functional recovery. After TBI, 13 proteins were up-regulated in the brain cortex, while basic fibroblast growth factor (bFGF) and fibroblast growth factor-binding protein (FGF-BP) were down-regulated after ghrelin treatment. It is known that bFGF can induce angiogenesis in the brain and accelerate wound healing, which can be further enhanced by FGF-BP. Based on the previous studies, it is hypothesized that the exogenous ghrelin curing TBI might cause the closure of bFGF and FGF-BP functions on wound healing, or ghrelin might exert the neuroprotective effects by competitively inhibiting bFGF/FGF-BP-induced neovascularization. Whether the combinational administration of ghrelin and bFGF/FGF-BP can enhance or weaken the therapeutic effect on TBI requires further research.

## Introduction

Traumatic brain injury (TBI) is a major public health concern and carries a high morbidity and mortality (Aarabi and Simard, 2009). Globally, approximately 10 million TBI cases are reported each year. Patients with TBI typically show various symptoms including headaches, dizziness, imbalance, vertigo, fatigue, changes in sleep pattern, neuropsychiatric symptoms, cognitive impairments, and even death (McCauley et al., 2005). Although advances in research and improved neurological intensive care have been achieved in recent years, the clinical outcome of patients with severe TBI remains poor, emphasizing the need for further research and improvement in care.

Ghrelin, a 28-amino acid peptide secreted mainly in the stomach, is a neuroendocrine hormone and an endogenous ligand for growth hormone secretagogue receptor (GHS-R) (Kojima et al., 1999). Additionally, ghrelin is slightly produced in the central nervous system, such as in the arcuate nucleus of the hypothalamus, ependymal layer of the third ventricle, dorsomedial nucleus, paraventricular nucleus, ventromedial nucleus and the ventral tegmental area (Abizaid, 2009). It has been suggested that ghrelin is protective against TBI (Bansal et al., 2010; Wei et al., 2015). Ghrelin-treated mice show significant neuroprotection following TBI including preservation of neurons, inhibition of neuronal apoptosis, and prevention of blood–brain barrier breakdown (Lopez et al., 2011, 2012). However, the molecular mechanism behind the protective role of ghrelin against TBI is unclear.

A protein plays its role in organisms via affecting other proteins, which forms a protein–protein interaction network. In the present study, in order to reveal the molecular mechanism of ghrelin against TBI, we used an advanced antibody array technology that has the advantages of being amenable to high-throughput screening and rapid parallel detection of multiple proteins.

## Materials and Methods

### Animals

Sprague-Dawley rats weighing 300–350 g were provided by the Chinese People’s Liberation Army Medical Center Experimental Animal Center [SCXK- (Army) -2007-004]. Experimental procedures were approved by the Animal Ethics Committee of the Academy of Military Medical Sciences.

### TBI Model and Ghrelin Treatment

Sixty rats were anesthetized with 50% chloral hydrate, vertical incisions were made over the cranium and the periosteums were stripped off. Using a surgical drill, a burr hole, 5 mm in diameter, at the junction 5 mm posterior to the coronal suture and 5 mm to the right of the sagittal suture, was created to expose the dura mater in each rat. Random 30 rats were fixed in the electron cortical contusion impactor (eCCI 6.3, Custom Design and Fabrication, Richmond, VA, United States). A strike was made onto the dura mater at a 3 mm depth and 5 m/s rate. The incisions in all rats were sutured. Among 30 stricken and the other 30 not stricken rats, 15 individuals were randomly chosen from each group to be treated with intravenous ghrelin (Phoenix Pharmaceuticals, Burlingame, CA, United States) at 20 μg/kg dose 30 min after sutured.

### Evaluation of Neurological Severity Scale

At 6, 24, 48 and 72 h after TBI, neurological functional deficits of all animals were evaluated according to the Neurological Severity Scale (NSS). The neurological scoring method was used based on the literature (Huang et al., 2005). This NSS includes motor function, sensory function, balance capacity and reflexes (details are included in **Table 1**). The maximum neurological score was 18 points. A score of 13–18 points denoted severe injury, 7–12 points showed moderate injury, and 1–6 points indicated mild injury. Neurological scores were expressed as mean ± standard deviation (SD).

**Table 1:** Neurological severity scale content.

Signs	Description score	
Motor function	When held by tail: (1) Forelimb flexion; (2) Hindlimb flexion; (3) Angle of head moving basing on the vertical axis greater than 10° in 30 s	3
	Moving on horizontal surface: (1) Normally walking; (2) Can not straight walk; (3) Constant circling toward paretic side; (4) Tumbling toward paretic side	3
Sensory function	(1) Orienteering test (vision and tactile); (2) Proprioception test (deep feeling)	2
Balance capacity	(1) Staying and walking parallel on the beam; (2) Staying on the beam, with hindlimb hanging; (3) Staying on the beam, with hindlimbs hanging or circling for more than 60 s; (4) Falling off with attempt to stay on the beam for more than 40 s (5) Falling off with attempt to stay on the beam for more than 20 s (6) Falling off within 20 s with attempt to stay on the beam	6
Reflexes	(1) Auricular reflex (head shaking when stimulating the ear canal); (2) Corneal reflex (blinking when touching the cornea with cotton swab; (3) Startle reflex (moving when hearing short and sharp voice); (4) Epilepsy or dystonia	

### Histological Examination

At 72 h after TBI, the animals were abdominally anesthetized, and were perfused with cold normal saline. After removal from the cranial cavity, the cortices were isolated from the brain, and were cut into two parts. One part was fixed in 4% paraformaldehyde. The cortex samples were embedded in paraffin, then were cut into 4 μm sections and stained with hematoxylin and eosin (HE). Histology was observed in a blinded manner using a BX53 light microscope (Olympus Corporation, Japan) at 400× magnification and documented with photographs.

### Antibody Array Assay

The second part of the cortices was ground to obtain protein extracts for measurement using Rat Cytokine Antibody Arrays (RayBio Rat Cytokine Antibody Array 3 and 4, glass series; RayBio Growth Factor Antibody Array, membrane series. RayBiotech, Norcross, GA, United States), which simultaneously detect 75 cytokines (details in **Supplementary Table S1**), according to the manufacturer’s instructions. Briefly, protein extracts was diluted to 500 μg/ml with blocking buffer and added to the array pools printed with 75 corresponding anti-cytokine antibodies for overnight incubation. After washing, a biotin-conjugated anti-cytokine mix was incubated with the pools for 2 h. Finally, the glass series arrays were performed with Cy3-conjugated streptavidin, while the membrane series arrays were incubated with HRP-conjugated streptavidin for a further 2 h. After the experimental procedure, the glass slides were scanned to detect the fluorescent signals of microarrays using an InnoScan 300 Microarray Scanner (Innopsys, France). The membrane arrays were exposed after HRP catalyzing chemiluminescent solution using an ImageQuant LAS4000 Scanner (GE Healthcare, Waukesha, WI, United States). The signal values were read and normalized, using an internal positive control, by the RayBiotech analysis tool, which is specifically designed to analyze the data of the Rat Cytokine Antibody Array 3 and 4 and Growth Factor Antibody Array.

### ELISA Identification

In order to validate the results from the antibody array, ELISA kits (RayBiotech, Norcross, GA, United States) were used to measure significant cytokines, according to the manufacturer’s instructions. Briefly, protein extracts of cortex samples were incubated in plates coated with capture antibody overnight at 4°C. The plates were washed and a biotin-conjugated detection antibody was added into the plates for incubation (2 h, room temperature) to combine with corresponding proteins. HRP-conjugated streptavidin was added to the plates and allowed to incubate for 45 min. TMB reagent was added and allowed to incubate for 30 min before the reaction was stopped with sulfuric acid. Immediately, the optical density was measured via an ELx800NB microplate reader (BioTek, Winooski, CT, United States) at 450 nm wave length.

### Statistical Analysis

All data were statistically analyzed by Student’s *t*-test using SPSS v.17.0 (SPSS, Inc., Chicago, IL, United States) and are presented as mean ± SD. Differences were considered significant if two-sided *p*-values were less than 0.05 (*p* < 0.05). In addition, fold change values were given to indicate the relative expression levels of cytokines. The sample size were calculated basing on the proteins data of antibody array altered in TBI and ghrelin groups from the pilot study using EmpowerStats software with two-tailed test. With a power of 80% and a significance level of 5%, 15 rats per group were needed.

## Results

### Post-TBI Effect of Ghrelin on the NSS

**FIGURE 1:**
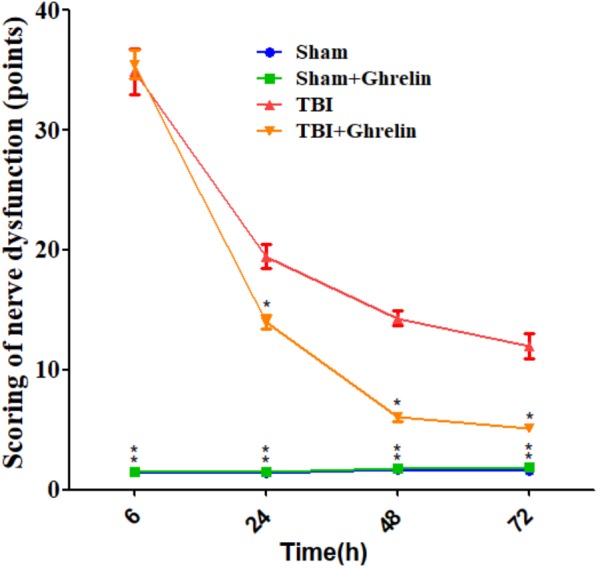
The NSS results of nerve dysfunction in rats. The NSS were performed at 6, 24, 48, and 72 h after TBI. Data are presented as means ± SD. ^∗^*p* < 0.05 versus TBI group at the same time point. *n* = 15 in each group.

The effect of ghrelin on improved nerve function was evaluated according to the NSS. As shown in **Figure 1**, NSS scores were significantly increased after TBI at different time points, indicating the successful establishment of the TBI model. However, ghrelin treatment markedly decreased NSS scores at 24, 48, and 72 h. For example, symptoms including contralateral forelimb flexion, tilting to the contralateral side and other neurobehavioral changes caused by TBI were significantly alleviated by ghrelin treatment.

### Effect of Ghrelin on Histological Improvement

**FIGURE 2:**
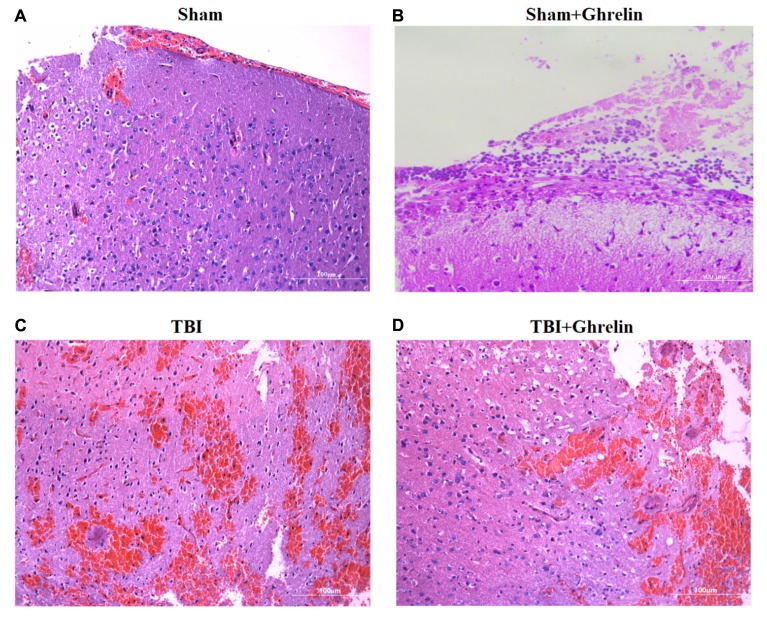
Ghrelin-induced morphological improvement of the cortex after TBI. HE staining of brain cortices 72 h after TBI was performed. **(A)** HE staining (×400) of the sham-operation area of the sham group. **(B)** HE staining (×400) of the sham-operation area of of the sham + ghrelin group. **(C)** HE staining (×400) of the injured brain area of the TBI group. **(D)** HE staining (×400) of the injured brain area of the TBI + ghrelin group.

Hematoxylin and eosin staining of brain cortices at 72 h after TBI showed extensive edema, neuronal necrosis, karyolysis and vacuolar changes, and widened intercellular gaps. In the ghrelin group, ghrelin treatment obviously reduced damage, including moderating neuronal degeneration, reducing the extent of dark nuclear staining, a lack of shrunken cells, no significant formation of intercellular gaps, and less edema (**Figure 2**).

### Altered Cytokine Levels in the TBI Group

**FIGURE 3:**
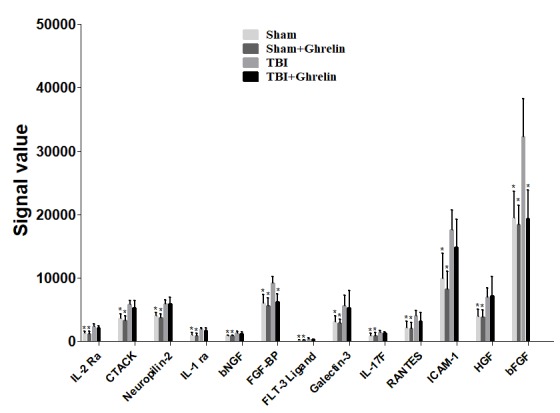
Altered factors after TBI. Thirteen proteins were found to be significantly increased after TBI, but only FGF-BP and bFGF were decreased after ghrelin treating TBI. The signal values from 13 proteins are shown by histograms to exhibit their expression levels. ^∗^*p* < 0.05 versus TBI group. Data are presented as means ± SD. *n* = 15 in each group.

Statistical analysis results showed that, compared to sham group, IL-17F, IL-1 ra, RANTES, Galectin-3, ICAM-1, basic fibroblast growth factor (bFGF), HGF, IL-2 Ra, FLT-3 Ligand, CTACK, fibroblast growth factor-binding protein (FGF-BP), bNGF, and Neuropilin-2 were significantly up-regulated in the TBI group (**Figure 3**, *p* < 0.05, detailed data in **Supplementary Data Sheet S1**).

### Effect of Ghrelin on Protein Expression After TBI

**FIGURE 4:**
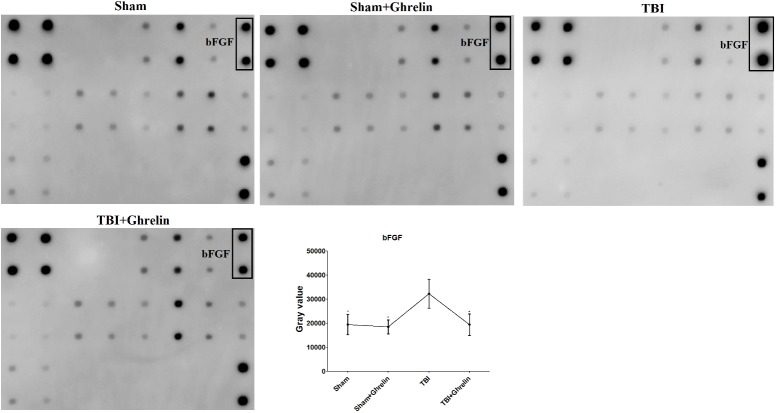
Antibody array profiles and expression levels of bFGF among the four groups. bFGF was detected by a RayBio Growth Factor Antibody Array, which is a membrane series array. bFGF location in array is encircled by black boxes. The signal values of bFGF among the four groups is shown by line graphs. ^∗^*p* < 0.05 versus TBI group. Data are presented as means ± SD. *n* = 15 in each group.

**FIGURE 5:**
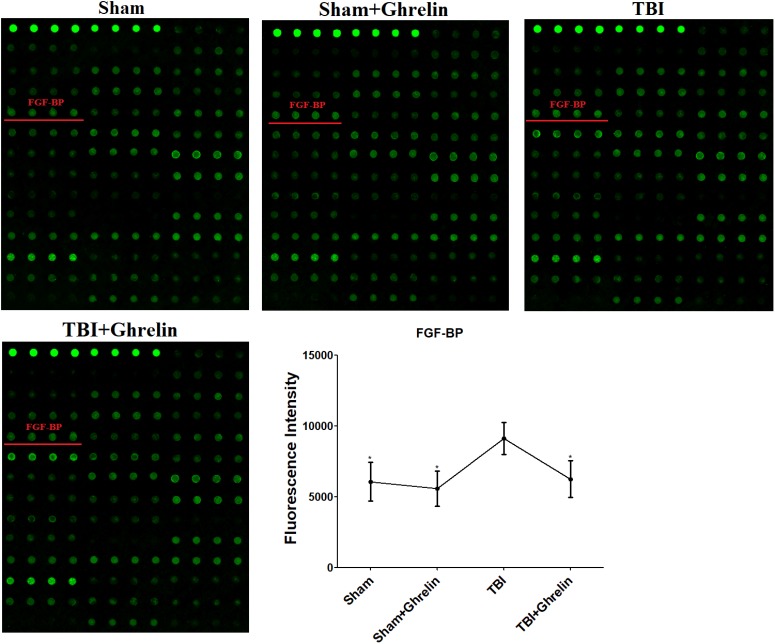
Antibody array profiles and expression levels of FGF-BP among the four groups. FGF-BP was detected with a RayBio Rat Cytokine Antibody Array 4, a glass series array. FGF-BP location in the array is labeled by red underscores. The signal values of FGF-BP among the four groups is shown by line graphs. ^∗^*p* < 0.05 versus TBI group. Data are presented as means ± SD. *n* = 15 in each group.

To reveal the molecular mechanism of ghrelin treating TBI, the proteins expressed differentially between the TBI and ghrelin groups were statistically analyzed. As a result, among the cytokines increased in the TBI group, bFGF and FGF-BP were obviously decreased after treatment with ghrelin. As shown in **Figure 4**, the gray signal spots of bFGF in the Growth Factor Antibody Array in the TBI group were larger than in the sham group. Similarly, as shown in **Figure 5**, FGF-BP, in the Cytokine Antibody Array 4, displayed brighter fluorescent signals. These results represented bFGF and FGF-BP levels were increased in the TBI group, and were decreased in ghrelin group.

### ELISA Validation Results

**FIGURE 6:**
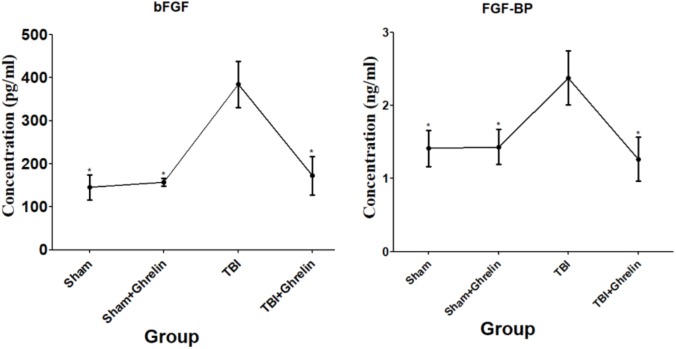
ELISA validation for bFGF and FGF-BP. The proteins participating in the molecular mechanism of ghrelin against TBI bFGF and FGF-BP were chosen for validation by ELISA. The concentrations of bFGF and FGF-BP obtained from ELISA are shown by line graphs. ^∗^*p* < 0.05 versus TBI group. Data are presented as means ± SD. *n* = 15 in each group.

In order to validate the altered protein expression affected by ghrelin, ELISA was performed for bFGF and FGF-BP. Consistently, the ELISA results showed that bFGF and FGF-BP were up-regulated after TBI, and were down-regulated in the ghrelin treatment group (**Figure 6**). These results were in accordance with the results of the antibody array, and certify further the effect of ghrelin on bFGF and FGF-BP expression.

## Discussion

Traumatic brain injury with neurological functional impairment has high rates of mortality and morbidity, can cause complicated metabolic, physiologic, and cellular responses, and, in particular, a marked inflammatory response. In the present study, we utilized traditional methods such as NSS evaluation and histological examination to authenticate a TBI model. Additionally, antibody array technology revealed that TBI could result in elevated expressions of IL-17F, IL-1 ra, RANTES, Galectin-3, ICAM-1, bFGF, HGF, IL-2 Ra, FLT-3 Ligand, CTACK, FGF-BP, bNGF and Neuropilin-2. As reported previously, among these biomarkers, IL-17F, IL-1 ra, RANTES, Galectin-3, ICAM-1,bFGF,HGF, IL-2 Ra, bNGF and Neuropilin-2 had been found to exhibit raised levels in brain injury (Kumon et al., 1993; Isaksson et al., 1997; Gabellec et al., 1999; Lenzlinger et al., 2001; Shimamura et al., 2007; Svetlov et al., 2012; Zhuang and Li, 2013; Shen et al., 2016; Albert et al., 2017; Li et al., 2017), which further demonstrated the successful establishment of our TBI model and revealed the molecular mechanism promoting TBI. Moreover, FLT-3 Ligand, CTACK and FGF-BP were found to be increased after TBI, implying the complexity of TBI pathogenesis. Bioinformatics analysis of these factors showed the most enriched Gene Ontology (GO) terms were “response to external stimulus,” “cellular response to stimulus,” “response to stimulus.” TBI occurs from external trauma, which will activate these proteins in order to regulate the cellular response to this stimulus.

More importantly, in the present study, ghrelin treatment exhibited evident improvement of TBI symptoms. The improvement was corroborated by the results of NSS evaluation and histological examination. Ghrelin is a 28-amino acid orexigenic peptide, and has been found to have neuroprotective effects in several models of neurological diseases. Relevant to the neuroprotective mechanism, Sun et al. (2016) discovered ghrelin attenuates brain injury in septic mice by increasing the expression of p-Akt and Bcl-2 and decreasing Bax expression. Previous study (Qi et al., 2014) reported that ghrelin protects rats against traumatic brain injury through up-regulation of UCP2. However, the mechanism by which ghrelin acts against TBI may be complex due to the interplay of proteins. The present study is the first to investigate comprehensively the mechanism of ghrelin against TBI by employing an antibody array. As a result, we found 13 factors that were up-regulated in the brain cortex after TBI, and, among these factors regulating TBI progression, bFGF and FGF-BP were obviously decreased after ghrelin treatment.

Basic fibroblast growth factor, as one of growth factors which are important mediators for neurogenesis, is a potent mitogenic factor for neural stem cells. Some studies have shown that administration of bFGF could reduce focal ischemia-induced infarct size, improve sensorimotor function, and promote functional recovery of rats who were subjected to motor cortex lesions (Li and Stephenson, 2002; Monfils et al., 2005). Moreover, elevated bFGF expression levels have been found during brain development and various forms of brain insult, suggesting that elevation of bFGF may play a key role in initiating nerve regeneration and scar production to respond the cellular wounding (Logan et al., 1992; Shetty et al., 2005). FGF-BP is a secreted protein that reversibly and non-covalently binds these FGFs to protect them from degradation and to enhance their biological activity. Some studies have reported that FGF-BP is up-regulated during the initial healing phase, such as a FGF-BP upregulation after surgical wounding of human skin grafts or of mouse skin within a few hours, but a return to control levels after 2 days with wound closure, and exogenous FGF-BP increased the activity of bFGF to stimulate neurite outgrowth and accelerated wound healing by enhancing bFGF-mediated angiogenesis (Kurtz et al., 2004; Beer et al., 2005; Tassi et al., 2007). Similarly, the present study showed the levels of bFGF and FGF-BP were elevated during TBI, implying bFGF and FGF-BP secretion might be activated by the organism to promote wound healing and resist TBI. Then, after ghrelin treatment for 72 h, the levels of bFGF and FGF-BP were reduced, suggesting that ghrelin might effectively treat TBI, causing the closure of bFGF and FGF-BP effects on wound healing by down-regulating bFGF and FGF-BP. As known, bFGF and FGF-BP involve into the wound healing process by neovascularization of wounds. However, Baiguera et al. (2004) found that ghrelin could inhibit bFGF-mediated angiogenesis of rat brain microvascular endothelial cells, hinting exogenous ghrelin probably promotes the repair of injured brain tissues by another pathway and competitively inhibiting bFGF/FGF-BP-induced neovascularization. Therefore, it is unclear whether the relationship of Ghrelin with bFGF and FGF-BP is causative or competitive, which might need further identification.

## Conclusion

We show that ghrelin attenuated brain injury and promoted brain functional recovery by reducing the levels of bFGF and FGF-BP. However, it has not been identified whether the relationship of Ghrelin with bFGF and FGF-BP on TBI treatment is causative or competitive, which will be researched soon.

Moreover, if the combinational administration of ghrelin and bFGF/FGF-BP can enhance the therapeutic effects in TBI, this would be a great stride in the treatment of TBI.

## Author Contributions

XS and XJ conceived and designed the experiments and analyzed the data. XS, QH, SC, QW, PX, and XJ performed the experiments. XS wrote the paper.

## Conflict of Interest Statement

The authors declare that the research was conducted in the absence of any commercial or financial relationships that could be construed as a potential conflict of interest.

## Supplementary Material

TABLE S1 Maps of RayBio antibody arrays.

DATA SHEET S1 Antibody array data of differential proteins.
