# Supplementary material for: Ghrelin Ameliorates Traumatic Brain Injury by Down-Regulating bFGF and FGF-BP
Source: Front Neurosci. 2018 Jul 5;12:445. doi: 10.3389/fnins.2018.00445 (PMC6041414; doi:10.3389/fnins.2018.00445)

RayBio Growth Factor Antibody Array Map


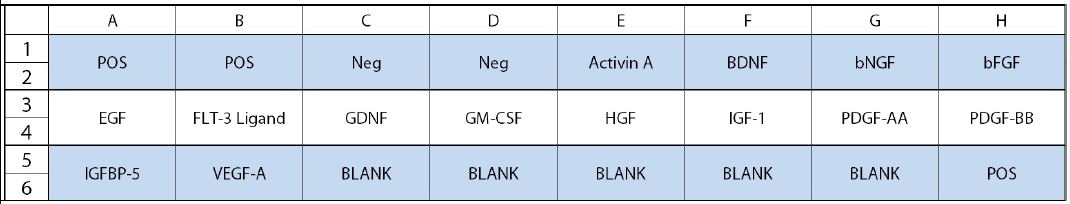


RayBio Rat Cytokine Antibody Array 3 Map


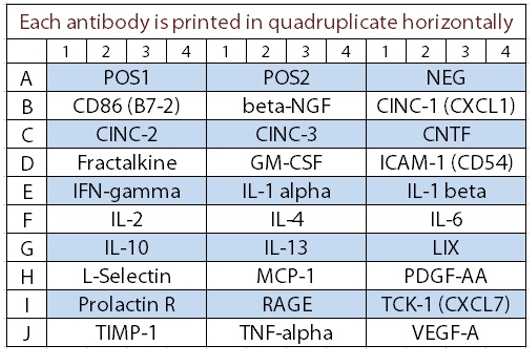


RayBio Rat Cytokine Antibody Array 4 Map


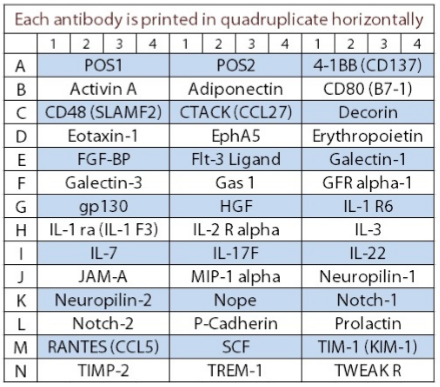

Supplement: TABLE S1 — Maps of RayBio antibody arrays. [file Table_1.DOCX]
